# The Story of the Insane from Year to Year

**Published:** 1901-12-07

**Authors:** 


### THE STORY OF THE INSANE FROM YEAR TO YEAR.

(Thirteenth Series.)

### LANCASHIRE COUNTY ASYLUM AT WHITTINGHAM.

Here the numbers on January 1st, 1899, were 1,933; on December 31st they had fallen to 1,830. There were 326 first admissions, 17 readmissions, 115 recoveries, 16 discharged relieved, 16 discharged not improved, and 198 deaths. The average number resident during the year was 1,937, and the total number under treatment was 2,271. The recoveries were 33 92 per cent. of the admissions, and the deaths were 10·22 per cent. of the average number resident. Of the year's deaths, 50 were caused by cerebral and spinal diseases, 27 by pnuemonia, 6 by bronchitis, 4 by gangrene of the lung, and 63 by consumption. That asylum pest, ulcerative colitis, was responsible for 5 of the deaths. We learn from Table XII. that of the year's admissions, domestic trouble was the assigned cause of the insanity in 8 cases; adverse circumstances in 17, mental anxiety in 10, religion in 4, intemperance in drink in 67, and hereditary influence in 70. In 49 no cause was known. Dr. Percival refers to the high mortality from phthisis, and he suggests that a small hospital should be built for consumptives. In the meantime an attempt is being made to isolate these patients. Regarding the colitis we failed to find any reference. A block for the treatment of 200 acute cases has been opened, and we shall watch anxiously for the 60 per cent. of recoveries predicted. Dr. Perceval regrets that expense stood in the way of putting wood block flooring throughout this building; but it is difficult to see how this could have been more expensive than an ordinary floor unless a great deal of making-up ground had to be done. No doubt Dr. Percival saw that a six-inch layer of concrete was placed over the ground in any case, and if he works the problem out he will probably find that the blocks would have been as cheap as sleeper walls, joists, and floor boards. The average weekly cost per head was 8s. 5¾d.

### GLAMORGAN COUNTY ASYLUM AT BRIDGEND.

On January 1st, 1899, this asylum contained 1,575 patients, and on December 31st the number had risen to 1,589. There were 389 first admissions, 65 readmissions, 109 recoveries, 51 discharged relieved, and 150 deaths. The total number of persons resident during the year was 2,011, and the average number resident was 1,575. The percentage of recoveries calculated on the admissions was 25·1 per cent. This is much lower than the average of county asylums, and it is also lower than the Glamorgan average. The percentage of deaths calculated on the average number resident was 9·5 which is about the county asylum average. Of the year's deaths 54 were caused by cerebral and spinal diseases, 22 by phthisis, 11 by pneumonia, 1 by pleurisy, 1 by bronchitis, 20 by Bright's disease, and 1 by enteritis. Of the admissions it is supposed that moral causes—domestic trouble, mental anxiety, religion, etc.—were responsible for the insanity in 104 instances, intemperance in drink in 114, hereditary influence in 132, and in 71 there was no cause known. The asylum is something more than full, there being 38 patients in excess of the proper accommodation, and it is proposed to relieve this overcrowding by the erection of temporary buildings or by boarding out a number of patients in other asylums. It is certain that the first course is an expensive one, and it is equally certain that the second one is a broken reed to lean on. Dr. Pringle has inaugurated a scheme of good-conduct pay, and he hopes "it will prove as great an encouragement to the well-doers as a deterrent and check to the thoughtless." All these things are helps to the good management of an asylum, and they are too often overlooked by committees and superintendents. The nursing classes have been continued and a large proportion of the staff hold the Medico-Psychological Nursing Certificate; but it would seem that 32 per cent. of the attendants and nurses have had less than one year's service. It is significant of the present dearth of young medical men that Dr. Pringle advertised for an assistant medical officer and did not get a reply "The committee desire to recognise the able and faithful interest taken in every department of the asylum by their esteemed medical superintendent." What profitable occupation farming is in Glamorgan! The asylum had a balance of £548 on the year's working. The average weekly cost per head was 8s. 8½d.

